# Long Non-Coding RNA RFPL3S Functions as a Biomarker of Prognostic and Immunotherapeutic Prediction in Testicular Germ Cell Tumor

**DOI:** 10.3389/fimmu.2022.859730

**Published:** 2022-05-20

**Authors:** Jie Guo, Shuang Wang, Zhenzhen Jiang, Le Tang, Zhizhong Liu, Jian Cao, Zhaolan Hu, Xiao Chen, Yanwei Luo, Hao Bo

**Affiliations:** ^1^ National Institution of Drug Clinical Trial, Xiangya Hospital, Central South University, Changsha, China; ^2^ China National Clinical Research Center for Geriatric Disorders, Xiangya Hospital, Central South University, Changsha, China; ^3^ Medical Research Center and Clinical Laboratory, Xiangya Hospital, Central South University, Changsha, China; ^4^ Department of Blood Transfusion, The Third Xiangya Hospital of Central South University, Changsha, China; ^5^ Reproductive Medicine Center, Maternal and Child Health Care Hospital of Hunan Province, Changsha, China; ^6^ Department of Urology, Hunan Cancer Hospital, The Affiliated Cancer Hospital of Xiangya School of Medicine, Central South University, Changsha, China; ^7^ Department of Anesthesiology, The Second Xiangya Hospital, Central South University, Changsha, China; ^8^ Department of Cosmedic, The First People’s Hospital of Changde City, Changde, China; ^9^ NHC Key Laboratory of Human Stem Cell and Reproductive Engineering, Institute of Reproductive and Stem Cell Engineering, School of Basic Medical Science, Central South University, Changsha, China; ^10^ Clinical Research Center for Reproduction and Genetics in Hunan Province, Reproductive and Genetic Hospital of CITIC-Xiangya, Changsha, China

**Keywords:** testicular germ cell tumor, long non-coding RNA, immunotherapy, biomarker, RFPL3S

## Abstract

The incidence of testicular germ cell tumor (TGCT) is currently on the rise worldwide, of which 15%-30% of patients have occur recurrence and metastasis. However, clinical methods for diagnosing TGCT and judging its prognosis remained inadequate. In this study, we aimed to explore the possibility of testis-specific long-chain non-coding RNA (lncRNA) Ret finger protein-like 3S (RFPL3S) as a biomarker for TGCT diagnosis, prognosis, and treatment response by reviewing the TGCT gene expression data in Gene Expression Omnibus (GEO) and The Cancer Genome Atlas (TCGA) databases. The cohort data and DNA methylation data of TGCT in TCGA were downloaded from TGCA, UCSC XENA, and GEO. The bioinformatic tools were used, including GEPIA2, Kaplan-Meier Plotter, LinkedOmics, UCSC XENA, Sangerbox Tools, GSCA, and Tumor Immune Dysfunction and Exclusion. Compared with normal testicular tissues, the RFPL3S expression was significantly reduced in TGCT, and was significantly negatively correlated with the patient’s Tumor, Node, Metastasis stage. Hypermethylation and low copy number of RFPL3S were present in TGCT, and low RFPL3S was associated with short disease-free and progression-free intervals. Silencing RFPL3S significantly enhanced the invasion ability and proliferation ability of TGCT cells as evaluated by Transwell and CCK-8 experiments. Additionally, RFPL3S expression was positively correlated with the infiltration of immune-activating cells such as B cells, CD8+ T cells, cytotoxic T cells, and natural killer cells, and negatively correlated with the infiltration of immunosuppressive cells such as Th17 and Th2. Higher RFPL3S expression was present in patients with immunotherapy benefits. In conclusion, we determined that the testis-specific lncRNA RFPL3S functioned as a tumor suppressor in TGCT and could be used as a prognostic predictor of TGCT, as well as a marker to predict the effect of TGCT immunotherapy.

## Introduction

Testicular germ cell tumor (TGCT) is the most common solid tumor in young males aged 20-40 years, and is one of the most common causes of death from solid tumors in males of this age (1). TGCT can be divided into two categories: seminoma and non-seminoma. The incidence is currently rising worldwide (1, 2), of which 15%-30% of patients have recurrence and metastasis. Such patients often have a poor prognosis (3). In recent years, researchers have found a variety of elevated genes expression in TGCTs. The novel germ cell markers, such as BOB1 and Prominin 1, were significantly up-regulated in seminoma (4). The expression of Aurora B expressed in spermatogonia and elevated in TGCTs (5). siRNA silenced the LIN28 gene in mice, and found that LIN28 plays an important role in the maintenance of seminoma (6). Houldsworth et al. found that Cyclin D2 and N-Myc were overexpressed in rat spermatogonia cells (7). Cyclin D2 is an early marker of carcinoma and plays an important role in the transformation of germ cell tumors (8). These studies demonstrate that TGCTs are caused by abnormal gene expression patterns, and genes related to mechanisms such as proliferation, pluripotency, and epigenetics, have different regulatory mechanisms in testicular tumor subtypes. The relationship between these gene targets and the pathogenesis of TGCT needs further study. Therefore, finding the biomarkers for early diagnosis and treatment response prediction in TGCT is particularly important.

These cancer-related RNA species are considered promising diagnostic, prognostic, and therapeutic targets, thus understanding their function in cancer development is becoming a major challenge (9). Studies have shown that long-chain non-coding RNAs (lncRNA) have good tissue and disease specificity and are promising biomarkers with clinical application (10, 11). High-throughput gene chip technology and RNA sequencing provide reliable means to find effective lncRNAs biomarkers. Yang et al. found that lncRNA MEG3 regulated the growth of TGCT through PTEN/PI3K/AKT signaling (12). Our previous study found that the expression of LINC00467 was positively correlated with the poor prognosis and pathological grade of TGCT, and LNC00467 could promote the migration and invasion of TGCT cells by regulating the expression of AKT3 and influencing AKT phosphorylation (1).

Ret finger protein-like 3S (RFPL3S) is an antisense transcript (exons 1-4) of the RFPL3 gene without apparent ORF and repetitive elements. Additionally, RFPL3S is specifically and highly expressed in testis compared with other human tissues (13). Reportedly, RFPL3S plays an important role in tumorigenesis. RFPL3S functioned as a transcriptional factor on the promoter of human telomerase reverse transcriptase to promote lung cancer growth (14–16), and acted as a potential stimulator of human immunodeficiency virus, type 1 (HIV-1) preintegration complex integration activity (17), suggesting that RFPL3S is involved in tumor growth and immune response. However, the role of RFPL3S in TGCT remains unknown.

Therefore, this study aimed to explore the possibility of testis-specific lncRNA RFPL3S as a biomarker for TGCT diagnosis, prognosis, and treatment response by reviewing the TGCT gene expression data in Gene Expression Omnibus (GEO) and The Cancer Genome Atlas (TCGA) databases. Here we revealed that the testis-specific lncRNA RFPL3S functioned as a tumor suppressor in TGCT and could be used as a prognostic predictor of TGCT, as well as a marker to predict the effect of TGCT immunotherapy.

## Materials and Methods

### Data Sources and Database

The cohort data and DNA methylation data of TGCT in TCGA were downloaded from UCSC XENA (https://xena.ucsc.edu/) (18). The data of GSE3218 and the TCGA single-cell sequencing data GSE120508 were downloaded from GEO. GSE3218 consists of 17 seminomas, 42 non-seminomas germ cell tumors, and 5 normal testis specimens (19). GSE120508 consists of approximately 6500 testicular cells from young adults (20).

GEPIA2 (http://gepia2.cancer-pku.cn/#index) was used to analyze the expression of RFPL3S in various tumors (21). The Kaplan-Meier Plotter database was used to analyze the relationship between RFPL3S and survival prognosis of TGCT patients (22). Based on TGCT data in TGCA, RFPL3S co-expression gene analysis, GO enrichment analysis, and GSEA enrichment analysis were performed using LinkedOmics (http://www.linkedomics.org/login.php) (23). Based on the methylation number of RFPL3S, UCSC XENA was used to analyze the correlation between RFPL3S and DNA methylation and copy number, and the relationship between RFPL3S methylation and copy number and prognosis of TGCT patients. Correlations between RFPL3S and stromal and immune scores were analyzed using Sangerbox Tools (http://www.sangerbox.com/tool). GSCA (http://bioinfo.life.hust.edu.cn/GSCA/#/), was used to analyze the correlation between RFPL3S expression, methylation, copy data and various immune cells (24). The responsiveness of immune checkpoint inhibitors was predicted using the Tumor Immune Dysfunction and Exclusion (TIDE) algorithm (25).

### Cell Culture and siRNA Transfection

NCCIT (CRL-2073) and Tcam-2 cell lines were purchased from the American Type Culture Collection (ATCC; Manassas, VA, USA). NCCIT cells and Tcam-2 cells were cultured in RPMI-1640 media plus 10% FBS and 1% penicillin at 37°C in 5% CO_2_. For knockdown RFPL3S, 10*10^5^ NCCIT and Tcam-2 cells were seeded in a 6-well plate. After the cells adhered, the transfection reagent Lip3000 mixed with RFPL3S siRNA or negative control (NC) siRNA (20 nM, Ribobio, Inc., Guangzhou, China) was added. After 6 hours of transfection, fresh complete medium was replaced, and subsequent experiments were carried out after culturing for 48 hours. The RFPL3S siRNA sequences were as follows: RFPL3S-siRNA-1: GTCACGTGTTTGCTTCACT, RFPL3S-siRNA-2: CCTTGATGTGTGAACAAAT. The nucleotide sequence of the non-target negative control (NC) was synthesized by Ribobio (Guangzhou, China).

### Quantitative Real Time PCR

qRT-PCR was accomplished as described in our previous described (26). After siRNA transfection, NCCIT and Tcam-2 cells were used for total RNA extraction by TRIzol reagent (Invitrogen). a First Strand cDNA Synthesis Kit (Roche, NJ, USA) was used to reverse transcribe RNA into cDNA. qPCR was accomplished on a Roche real-time PCR detection system (LightCycler480, Roche, USA). The following primers were used: RFPL3S, forward: 5′- GTCGTCAGAAATGAGGAGGAAGT-3′; reverse: 5′- TTGAAGTAGAAGAGAGGCATGGG-3′; ACTB, forward: 5-CTGAGGATGCGAGGTTCTGCTTG-3, reverse: 5-GTCACCGGAGTCCATCACGAT-3.

### Transwell Assay

Transwell assay was accomplished as described in our previous described (1). Briefly, NCCIT and Tcam-2 cells (2 × 10^4^/well in 200 µl of 2% FBS medium) were seeded on the upper transwell chambers with Matrigel (Corning, USA). The lower chamber was filled with 800 μl of 15% FBS medium. After culture for 48 h, the cells that had migrated through the membranes were fixed with paraformaldehyde and were stained with crystal violet. Five random fields were photographed in each group, and the number of invaded cells were counted under a microscope.

### Cell Counting Kit 8 Assay

CCK-8 assay was performed as described in our previous described (27). A CCK-8 kit was used for determining the cell viability of NCCIT and Tcam-2 cells after siRNA transfection according to the instructions.

### Statistical Analysis

Cell biology experimental data were obtained from at least three independent experiments. Statistical analysis was performed using SPSS 19.0 software (SPSS, Chicago, IL, USA). Differences between the two groups were assessed by Student’s t-test. A P value < 0.05 indicates a statistically significant difference.

## Results

### RFPL3S Was Downregulated in Testicular Germ Cell Tumor

**Figure 1:**
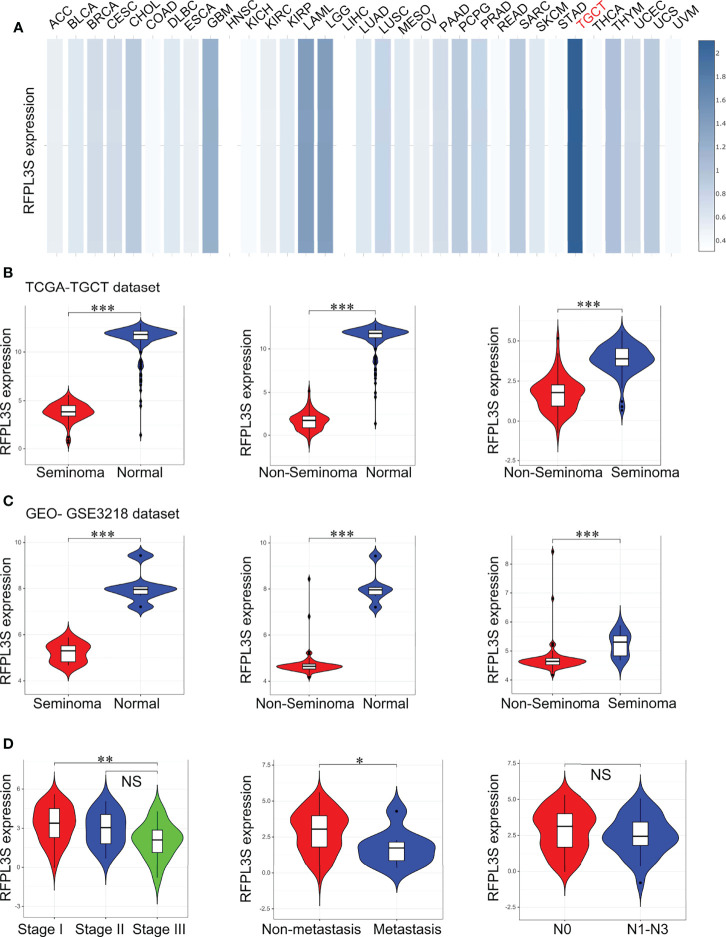
The association between RFPL3S and the clinicopathological characteristics of TGCT. **(A)** GEPIA2 online tool was used to analyze the expression of RFPL3S in various tumors. RFPL3S was highest expressed in TGCT (indicated by red color). **(B)** Analysis for the expression of RFPL3S in seminoma, non-seminoma, and normal tissues in TGCT cohort from TCGA. **(C)** Analysis for the expression of RFPL3S in seminoma, non-seminoma, and normal tissues in GSE3218 cohort. **(D)** Analysis for the association of the expression of RFPL3S with the clinicopathological characteristics (clinical stage, distant metastasis, and lymph node metastasis) of TGCT from TCGA. The TCGA TGCT cohort data was downloaded from the UCSC XENA database (https://xena.ucsc.edu/). The GSE3218 data was downloaded from the GEO database. Visualizing and interpreting cancer genomics data *via* the Xena platform. *P < 0.05, **P < 0.01, ***P < 0.001. NS, no significant.

At first, we analyzed the expression of RFPL3S in 33 tumors in the TCGA database through the TCGA pan-tumor data in the GEPIA database (28), and the results showed that RFPL3S was significantly highly expressed in TGCT (**Figure 1A**). We next used the TGCT cohort data in the TCGA database and the normal testicular gene expression data in the GTEX database to analyze the expression of RFPL3S in TGCT. We found that compared with normal testicular tissues, RFPL3S expression was significantly reduced in seminoma and non-seminoma (**Figure 1B**), and its expression in non-seminoma was lower than in seminoma (**Figure 1B**). The results of the TGCT cohort data (GSE3218) (19) was consistent with those in TCGA database (**Figure 1C**). In addition, we found that RFPL3S expression in TGCT was negatively correlated with patient’s TNM stage, and its expression in stage I was significantly higher than in stage III (**Figure 1D**). RFPL3S expression was associated with metastasis, and was lower in patients with distant metastasis than in those with non-metastasis (**Figure 1D**). However, RFPL3S was not correlated with lymph node metastasis in TGCT patients (**Figure 1D**).

### The Diagnostic and Prognostic Value of RFPL3S on TGCT

**Figure 2:**
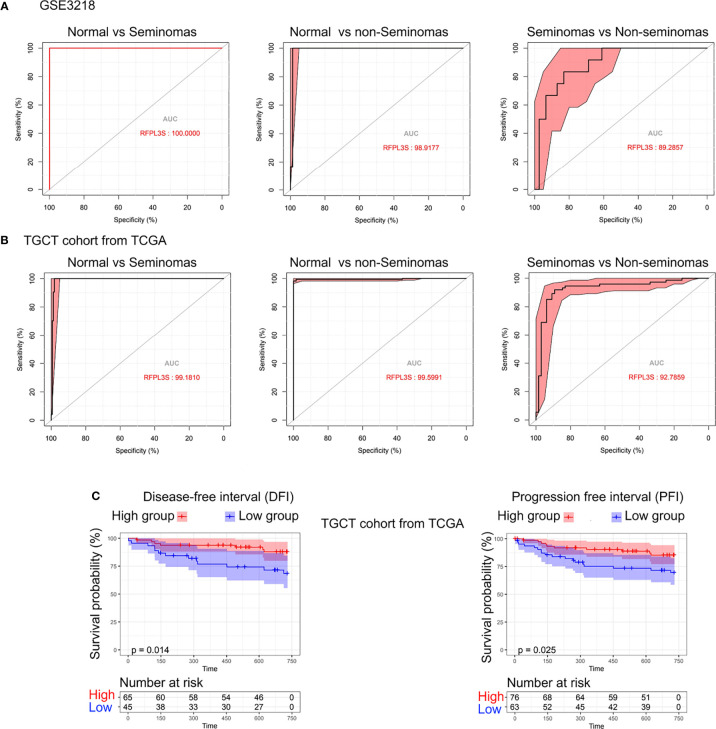
The diagnostic and prognostic value of RFPL3S in TGCT. **(A)** Receiver operating characteristic (ROC) curve evaluated the diagnostic value of RFPL3S in distinguishing normal (N) from seminoma (SEM), normal (N) from non-seminoma (NSEM), and non-seminoma (NSEM) from seminoma (SEM) in GEO GSE3218 cohort. **(B)** ROC curve evaluated the diagnostic value of RFPL3S in distinguishing normal (N) form seminoma (SEM), normal (N) from non-seminoma (NSEM), and non-seminoma (NSEM) from seminoma (SEM) in TGCT cohort from TCGA. **(C)** Correlation between RFPL3S and disease-free interval (DFI) and progression free interval (PFI) of TGCT cohort from TCGA. Patients with high RFPL3S expression had higher DFI and PFI.

Next, we studied the diagnostic and prognostic value of RFPL3S in TGCT based on the TCGA and GEO data (GSE3218). The RFPL3S expression could well distinguish normal tissues from seminoma, normal tissue from non-seminoma, and seminoma from non-seminoma (**Figures 2A, B**). In addition, we found a significant positive correlation between RFPL3S and disease-free interval (DFI) and progression free interval (PFI) in TGCT patients. Patients with high RFPL3S had higher DFI and PFI than those with low RFPL3S (**Figure 2C**). These data indicated that RFPL3S was a good TGCT tumor diagnosis and prognostic marker.

### RFPL3S Knockdown Inhibits Invasion and Proliferation of NCCIT and Tcam-2 Cells *In Vitro*

**Figure 3:**
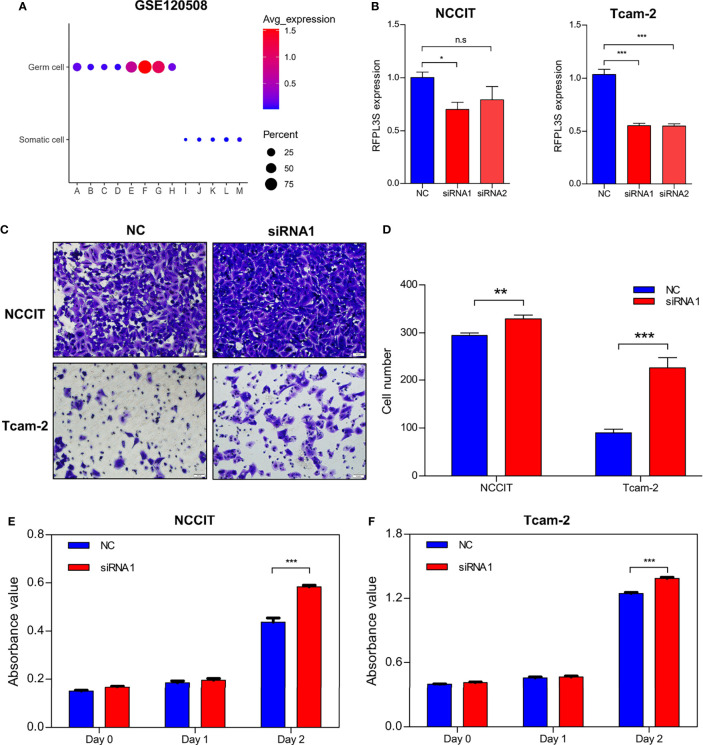
Knockdown RFPL3S inhibits invasion and proliferation of NCCIT and Tcam-2 cells. **(A)** The single-cell sequencing data of GSE120508 analyzes the expression of RFPL3S in different cell types, showing that RFPL3S mainly expressed in germ cell. (A: spermatogonial stem cells; B: spermatogonia in differentiation; C: primary spermatocytes; D: secondary spermatocytes; E: round spermatozoa; F: elongated spermatozoa; G: sperm 1; H: sperm 2; I: macrophages; J: endothelial cells; K: muscle-like cells; L: support Cell; M: mesenchymal cell.). **(B)** siRNA transfection knocked down RFPL3S in NCCIT and Tcam-2 cells. **(C, D)** Silencing RFPL3S significantly enhanced the invasion ability of NCCIT and Tcam-2 cells evaluating by Transwell assay. **(E, F)** Silencing RFPL3S significantly enhanced the proliferation ability of NCCIT and Tcam-2 cells evaluating by CCK-8 experiment. *P < 0.05, **P < 0.01, ***P < 0.001. n.s, no significant.

**Figure 4:**
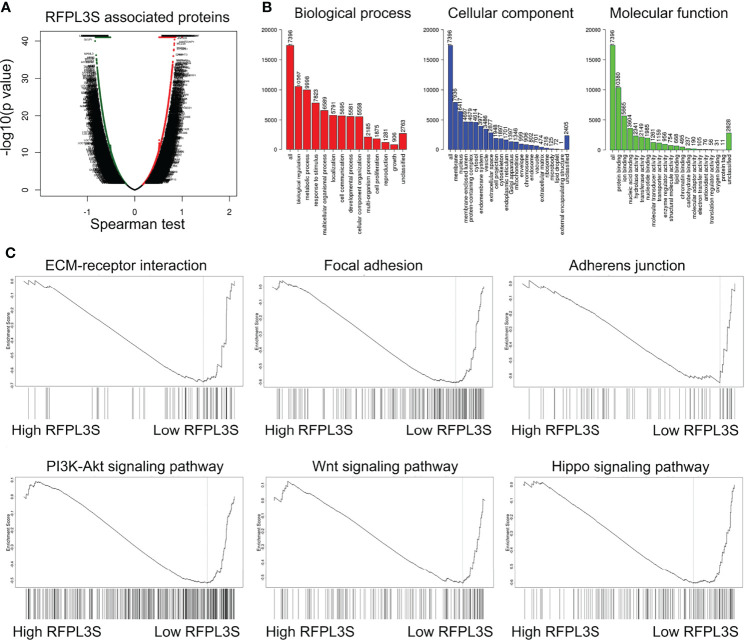
GSEA analysis for the regulatory signaling pathway of RFPL3S. Based on the TCGA TGCT expression profile data, LinkedOmics (http://www.linkedomics.org/login.ph) was used to analyze the co-expressed genes of RFPL3S **(A)**, GO enrichment analysis **(B)**, and GSEA enrichment analysis **(C)**.

We then used the testicular single cell sequencing data set GSE120508 to analyze RFPL3S expression in various types of cells (20), and observed that RFPL3S dominatingly expressed in germ cells (**Figure 3A**). Therefore, we speculated that RFPL3S played a role in TGCT cells rather than in the microenvironmental cells. To validate the function RFPL3S in TGCT, we designed two siRNA sequences to silence RFPL3S in NCCIT and Tcam-2 cells. The qPCR results showed that both siRNAs had a silencing effect, but the efficiency of siRNA1 was better than siRNA2 (**Figure 3B**). Silencing RFPL3S significantly enhanced the invasion ability (**Figures 3C, D**) and proliferation ability (**Figures 3E, F**) in NCCIT and Tcam-2 cells as evaluated by Transwell and CCK-8 experiments. We also found that the co-expressed genes and the downstream pathway of RFPL3S were associated with invasion and proliferation pathways, including ECM-receptor interaction, Focal adhesion, Adherent junction, PI3K-Akt signaling, Wnt signaling, and Hippo signaling (**Figures 4A–C**).

### The Association Between RFPL3S Expression, Methylation, and Copy Number in TGCT

**Figure 5:**
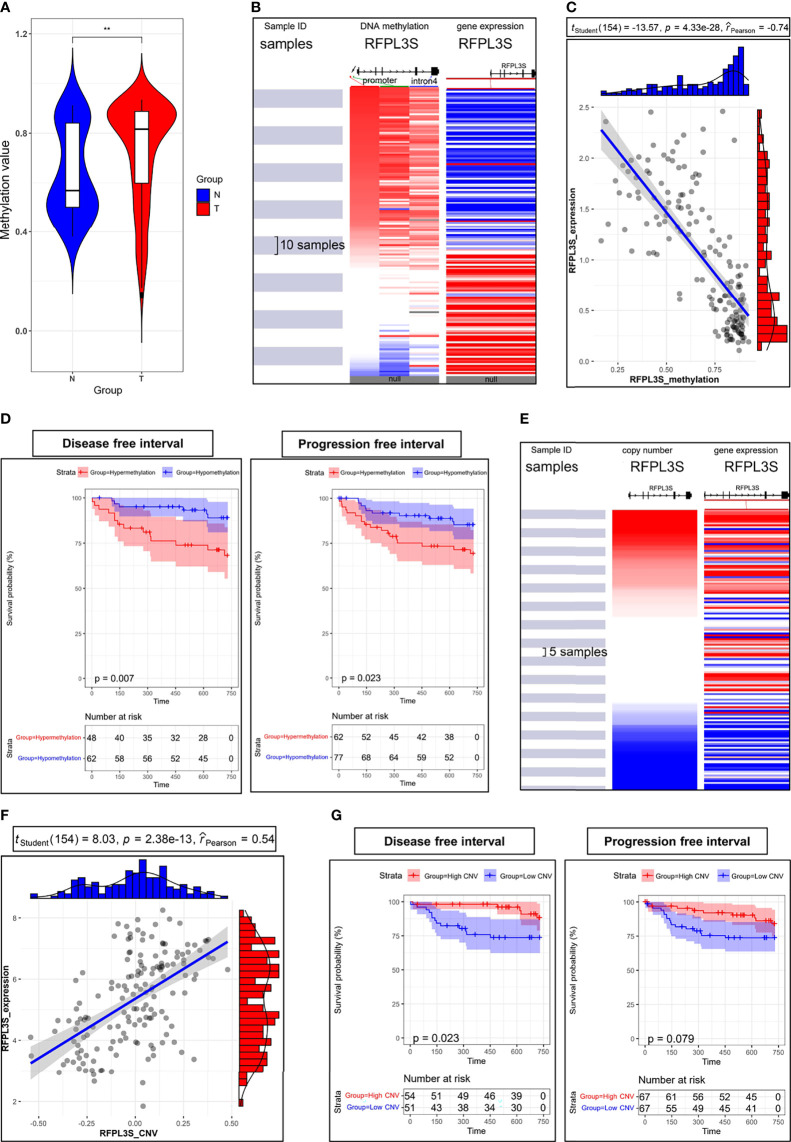
The association between RFPL3S expression, methylation, and copy number in TCGA TGCT cohort. **(A)** The methylation level of RFPL3S in normal testicular tissues (N) and TGCT (T) tissues. **(B)** The high methylation regions were located at promoter (indicated by red and green arrows) and intron 4 (indicated by blue arrow). **(C)** RFPL3S methylation level was significantly negatively correlated with the expression level. **(D)** Correlation between RFPL3S methylation level and disease-free interval (DFI) and progression free interval (PFI) of TGCT cohort from TCGA. Patients with high RFPL3S methylation level had lower DFI and PFI. **(E, F)** Copy number was significantly positively correlated with the expression of RFPL3S. **(G)** Correlation between RFPL3S copy number and disease-free interval (DFI) and progression free interval (PFI) of TGCT cohort from TCGA. Patients with high RFPL3S copy number had higher DFI and PFI. CNV, copy number various. The UCSC XENA database was used to download the methylation data, expression data, and prognosis data of RFPL3S from the TCGA TGCT cohort. **P< 0.01.

We next explored the upstream regulatory mechanism of RFPL3S in TGCT. We found that the methylation level of RFPL3S in TGCT tissue was significantly higher than that in normal testicular tissue (**Figure 5A**). RFPL3S had 3 highly methylated regions, 2 regions located at the promoter and 1 region located at the intron 4 (**Figure 5B**). RFPL3S methylation level was negatively correlated with its expression (**Figure 5C**). In addition, we found that the higher RFPL3S methylation was associated with lower DFI and PFI in TGCT patients (**Figure 5D**). The copy number of RFPL3S was positively correlated with its expression (**Figures 5E, F**), but was negatively associated with DFI and PFI in TGCT patients (**Figure 5G**).

### The Association of RFPL3S and TGCT Tumor Immunity

**Figure 6:**
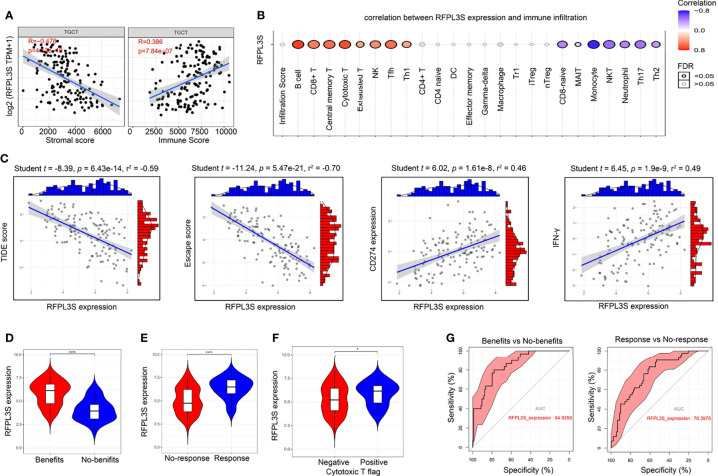
The association of RFPL3S and TGCT tumor immunity. Tumor Immune Dysfunction and Exclusion algorithm was used to predict the response of a single sample to predictive immune checkpoint inhibitors based on the TCGA TGCT expression profile data. **(A)** RFPL3S expression was negatively correlated with stromal score, and positively correlated with Immune score. **(B)** Correlation between RFPL3S expression and immune infiltrates in TGCT. **(C)** Correlation between RFPL3S expression and TIDE score, exclusion score, CD274 expression, and IFN-γ score in TGCT. **(D–F)** higher RFPL3S expression was present in the immunotherapy benefit, response, and cytotoxic T flag positive patients. **(G)** RFPL3S had a high diagnostic sensitivity and specificity for distinguishing immunotherapy benefits from no-benefits (AUC = 84.9359), and response from non-response (AUC = 76.3975). *P < 0.05, ****P < 0.0001.

The immune cell infiltration state in TGCT tissues has close correlation with immunotherapy response (29). We conducted immune cell analysis to explore the role of RFPL3S in TGCT tumor immunity. RFPL3S expression was negatively correlated with the matrix score of TGCT tissue, but positively correlated with the immune score (**Figure 6A**). RFPL3S expression was positively correlated with the infiltration of immune-activating cells such as B cells, CD8+T cells, cytotoxic T cells, and NK cells, whereas negatively correlated with the infiltration of immunosuppressive cells such as Th17 and Th2 (**Figure 6B**). Using the TIDE algorithm (25), we analyzed the correlation between RFPL3S expression and immunotherapy, and observed that the higher RFPL3S expression was associated with lower TIDE score and escape score in TGCT patients (**Figure 6C**), while was associated with higher the expression of the anti-tumor immune response markers CD274 and IFN-γ (**Figure 6C**). We found that Higher RFPL3S expression was present in patients with positive cytotoxic T flag or with immunotherapy benefits (**Figures 6D–F**), which had high diagnostic sensitivity and specificity (**Figure 6G**).

## Discussion

In recent years, the physiological functions of lncRNAs have been gradually elucidated, which are involved in various biological processes, such as chromatin remodeling, post-transcriptional regulation, protein translation and histone acetylation (30, 31). Abnormally expressed lncRNAs in human malignant tumors are closely related to tumor cell proliferation, invasion, and metastasis (1). RFPL3S covers the entire coding part of sense exon 2 of RFPL3. The normal function of RFPL3S is likely to post-transcriptionally regulate the sense RFPL (13). RFPL3S has been associated with Opitz syndrome, which is an inherited disorder characterized by midline defects including hypertelorism, hypospadias, lip-palate-laryngotracheal clefts and imperforate anus (13, 32). However, to our knowledge, there is no report on the role of RFPL3S in TGCTs. TGCTs including seminoma and non-seminoma, is characterized by slow progression and a good prognosis (33). At present, choosing an appropriate treatment modality is an important issue in the field of testicular tumor research (34). Studies have shown that classifying patients with tumor according to clinical information, including stage, grade, and molecular markers, and selecting appropriate treatment methods can improve patient prognosis and reduce adverse reactions related to surgery, radiotherapy, and chemotherapy (35). Therefore, combining large-scale databases such as TCGA, high-throughput GEO database, epidemiological, and prognostic databases to conduct in-depth mining of medical information is of great significance to guide TGCT treatment. This study is the first to discover the use of the expression profile of testis-specific lncRNA RFPL3S in differentiating seminoma from non-seminoma and prognostically predicting TGCT. High RFPL3S expression predicted a higher disease-free interval and progression free interval in TGCT patients.

Additionally, RFPL3S functioned as a tumor suppressor that significantly inhibited the invasion and proliferation of TGCT *in vitro*, and its expression was controlled by genetic factors (copy number variation) and epigenetic factors (DNA methylation). The analysis of downstream signaling pathways revealed that RFPL3S downregulation was associated with activated metastasis- and proliferation-related pathways, including extracellular matrix (ECM)-receptor interaction, Focal adhesion, Adherens junction, PI3K-AKT signaling, Wnt signaling, and Hippo signaling. The invasion and metastasis of tumor cells is a key factor that affects the prognosis of malignant tumors. The ECM is an important tissue barrier to prevent tumor metastasis. The main components, namely fibronectin and laminin, are linked to cell surface membrane integrin receptors, which determine the shape of cells and control cell differentiation and migration (36). Focal adhesion kinase plays an important role in cell cycle regulation, growth regulation, adhesion, cytoskeleton assembly, motility, and survival through a variety of signaling pathways (37). Studies have found that focal adhesion kinase is highly expressed in various tumors and plays an important role in the occurrence, development, invasion, and metastasis of tumors. Focal adhesion kinase may become a new target for tumor therapy (38).

In addition to its relationship with tumor cell proliferation and metastasis, we determined that higher RFPL3S expression was present in patients with immunotherapy benefit and response, and those positive with cytotoxic T flag. RFPL3S was associated with PI3K/AKT/mTOR, β-catenin/Wnt, and Hippo pathways, which have been associated with immunotherapy (39–46). These results suggest that RFPL3S may be an effective marker for predicting the efficacy of immunotherapy in patients with TGCT.

In conclusion, we determined that the testis-specific lncRNA RFPL3S functioned as a tumor suppressor in TGCT and could be used as a prognostic predictor of TGCT, as well as a marker to predict the effect of TGCT immunotherapy.

## Data Availability Statement

The original contributions presented in the study are included in the article/**Supplementary Material**. Further inquiries can be directed to the corresponding author.

## Author Contributions

All authors made a significant contribution to the work reported, whether that is in the conception, study design, execution, acquisition of data, analysis, and interpretation, or in all these areas. Took part in drafting, revising or critically reviewing the article. Gave final approval of the version to be published. Have agreed on the journal to which the article has been submitted, and agree to be accountable for all aspects of the work. All authors contributed to the article and approved the submitted version.

## Funding

This work is supported by Natural Science Foundation of Hunan Province (2020JJ5893, 2020JJ8001, 2021JJ41091), Scientific Research Fund of Hunan Provincial Health and Family Planning Commission (Correlation Analysis of Human Sperm Apoptosis and Outcome of IVF Pregnancy, No. 2015-145), the Fundamental Research Funds for Health Commission of Hunan Province (C2019073), the Changsha Municipal Natural Science Foundation (kq2014033), The Project of Changde Science and Technology Bureau of Hunan Province in China(2019S186).

## Conflict of Interest

The authors declare that the research was conducted in the absence of any commercial or financial relationships that could be construed as a potential conflict of interest.

## Publisher’s Note

All claims expressed in this article are solely those of the authors and do not necessarily represent those of their affiliated organizations, or those of the publisher, the editors and the reviewers. Any product that may be evaluated in this article, or claim that may be made by its manufacturer, is not guaranteed or endorsed by the publisher.
