# Supplementary figures and images for: Long Non-Coding RNA RFPL3S Functions as a Biomarker of Prognostic and Immunotherapeutic Prediction in Testicular Germ Cell Tumor
Source: Front Immunol. 2022 May 20;13:859730. doi: 10.3389/fimmu.2022.859730 (PMC9165694; doi:10.3389/fimmu.2022.859730)

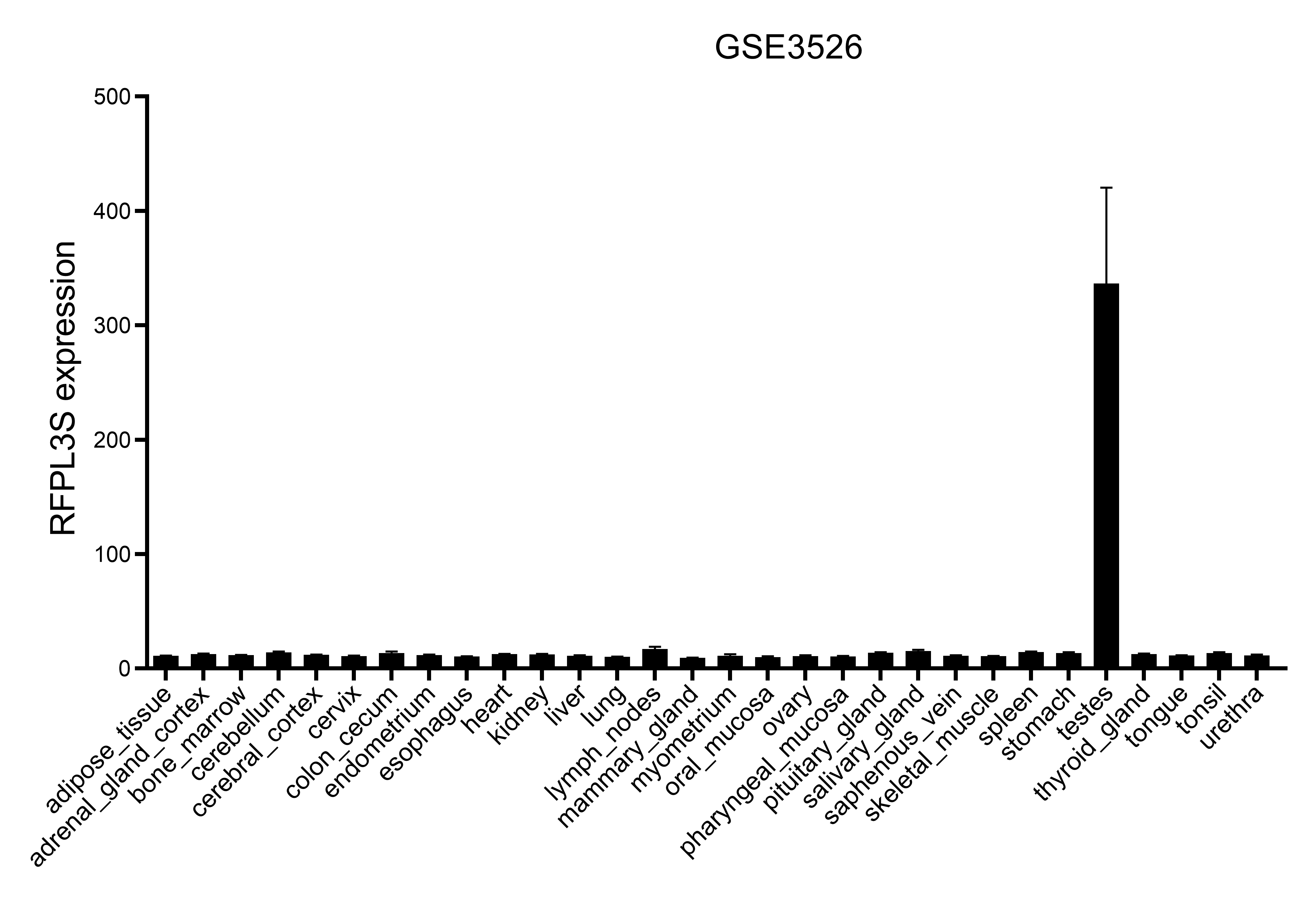

Supplement: Supplementary file 1 [file Image_1.tif]
